# County-level analysis reveals a rapidly shifting landscape of insecticide hazard to honey bees (*Apis mellifera*) on US farmland

**DOI:** 10.1038/s41598-019-57225-w

**Published:** 2020-01-21

**Authors:** Margaret R. Douglas, Douglas B. Sponsler, Eric V. Lonsdorf, Christina M. Grozinger

**Affiliations:** 1 Department of Environmental Studies & Environmental Science, Dickinson College, Carlisle, PA 17013 USA; 2 Department of Entomology, Center for Pollinator Research, Huck Institutes of the Life Sciences, Pennsylvania State University, University Park, 16802 PA USA; 3 Institute on the Environment, University of Minnesota, St Paul, MN 55108 USA

**Keywords:** Environmental impact, Agroecology

## Abstract

Each year, millions of kilograms of insecticides are applied to crops in the US. While insecticide use supports food, fuel, and fiber production, it can also threaten non-target organisms, a concern underscored by mounting evidence of widespread decline of pollinator populations. Here, we integrate several public datasets to generate county-level annual estimates of total ‘bee toxic load’ (honey bee lethal doses) for insecticides applied in the US between 1997–2012, calculated separately for oral and contact toxicity. To explore the underlying components of the observed changes, we divide bee toxic load into extent (area treated) and intensity (application rate x potency). We show that while contact-based bee toxic load remained relatively steady, oral-based bee toxic load increased roughly 9-fold, with reductions in application rate outweighed by disproportionate increases in potency (toxicity/kg) and extent. This pattern varied markedly by region, with the greatest increase seen in Heartland (121-fold increase), likely driven by use of neonicotinoid seed treatments in corn and soybean. In this “potency paradox”, farmland in the central US has become more hazardous to bees despite lower volumes of insecticides applied, raising concerns about insect conservation and highlighting the importance of integrative approaches to pesticide use monitoring.

## Introduction

Insects are the most diverse and abundant class of animals on earth, with an estimated 5.5 million species that dominate animal biomass in many ecosystems^1,2^. Given their ubiquity, it is not surprising that insect populations serve in key roles as both friend and foe to human societies. This is particularly true in agriculture, where farmers seek to manage populations of insect pests to produce essential food, fuel and fiber, a task in which insecticides play an important role. In the United States, agriculture accounts for ~57% of insecticide weight applied and ~85% of area treated, and so constitutes the single largest contributor to insecticide use^3,4^. However, since at least the 1960’s it has been widely recognized that insecticide application can also negatively affect non-target species, including populations of insect pollinators and natural enemies that serve to support crop production^5,6^. There has been a concomitant effort to reduce reliance on insecticides and/or minimize their non-target effects, including state and federal regulation, Integrated Pest Management (IPM) programs, and the development of alternative pest-management technologies and production/marketing systems such as organic farming^7^.

Nonetheless, studies suggest recent and widespread declines in insect abundance, diversity, and range^8–10^, and insecticide use has been identified as a likely contributor along with habitat loss, species introductions, and climate change^11^. In the US, declines have been documented in populations of several wild bee species, butterflies in Ohio and lowland California, and the migratory monarch butterfly^12–15^. Meanwhile, beekeepers sustain losses of >40% of their managed honey bee colonies annually^16^.

**Figure 1:**
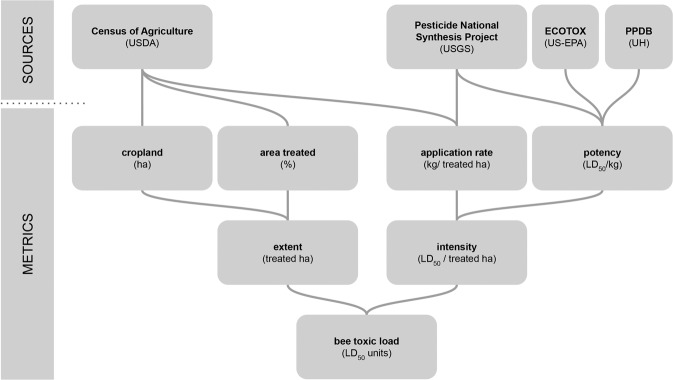
Schematic of bee toxic load, its components, and the data sources used to generate these values in this analysis. USDA = US Department of Agriculture, USGS = US Geological Survey, US-EPA = US Environmental Protection Agency, PPDB = Pesticides Properties Database.

To understand trends in pest management and potential impacts on target and non-target insect species, we argue it is important to disentangle four distinct dimensions of agricultural insecticide use, organized into two broad categories (Fig. 1), extent and intensity. At the landscape scale, the *extent* of insecticide use is a function of the area devoted to cropland as well as the proportion of that cropland treated with insecticides. Historically in the US, a majority of cropland has not been treated with insecticides^17^, likely because insect pest populations have not been problematic enough to justify treatment in low-value, large-acreage commodity crops such as wheat, soybean, and corn. For those fields that are treated, the *intensity* of insecticide use varies as a function of the application rate (kg active ingredient [AI]/ha) of products applied and the potency (toxicity/kg AI) of those products toward insects, typically measured via the LD_50_ (the dose required to kill 50% of a population). These factors jointly determine *insect toxic load*, the total number of insect lethal doses applied in a given area^18,19^. Because the honey bee (*Apis mellifera*) is the standard terrestrial insect in regulatory pesticide tests and so has the most comprehensive toxicity data available, thus far, calculations of this or similar metrics have depended on honey bee data^18,19^. We have followed this convention and will henceforth refer to this value as “bee toxic load.”

Past analyses of insecticide use on US farmland have arrived at divergent narratives of overall trends in insecticide use. For example, analyses based on farmer survey data from the US Department of Agriculture (USDA) show long-term national declines in the application rate of insecticides in corn and cotton crops, and corresponding reductions in the overall weight of insecticides applied^17,20^. A common interpretation of these trends is that introduction of transgenic insect-resistant (*Bt*) crops starting in the mid-1990s led to a reduction in insecticide application^17,20^. Conversely, data from the US Agricultural Census indicate that the extent of US insecticide use has *increased* since 1997, precisely in those regions dominated by *Bt* corn production^21–23^. The hypothesized cause of this increase in extent is the widespread adoption of neonicotinoid seed treatments^22,24^. The first analysis did not consider insecticide potency, while the second did not account for intensity. As these examples illustrate, the use of different metrics and data sources leads researchers to conflicting interpretations of overall trends in insecticide use and their associated causes.

Importantly, describing aggregate trends in pesticide use is not very meaningful without a consideration of the potency of diverse active ingredients^18,25^. In the US alone, there are more than 1000 pesticide active ingredients registered for use^26^, which vary by orders of magnitude in potency to target and non-target organisms^27^. Aggregate trends in application rate or total weight applied can mask important shifts in the use of different pesticide products over time. For insecticides, this is particularly problematic given evidence of a long-term trend toward development of active ingredients with increasing potency toward insects^28^.

Two recent analyses examined trends in bee toxic load at the national scale in Great Britian^18^ and the US^19^. Goulson *et al*.^18^ found that in Great Britain, from 1990 to 2015, the quantity (kg) of pesticide applied decreased, but the amount of treated agricultural land (ha) increased and the toxic load to bees increased 6-fold. DiBartolomeis *et al*.^19^ demonstrated that in the US, from 1992 to 2014, there was an overall increase in bee toxic load by 4-fold (calculated using honey bee contact LD_50_) or 48-fold (calculated using honey bee oral LD_50_). DiBartolomeis *et al*. further found that neonicotinoids in corn and soy crops were the primary drivers of this increase.

Building on these national analyses, the research presented here characterizes for the first time the spatial dimension of changes in bee toxic load, by estimating this value and its contributing elements of insecticide extent and intensity at the county scale. In the US, there is considerable regional variation in insecticide use patterns due to the large size of the country, the concentration of different crop production systems in particular areas, the divergent insecticide regimes associated with different crops, and regional variation in pest pressure^23,29,30^. Analyses at regional to local scales are vital to target insecticide mitigation efforts and to facilitate research relating spatial patterns in insecticide use to spatial patterns in important outcomes such as crop production, insecticide resistance, and pollinator decline. Differentiating components of insecticide extent and intensity further elucidates syndromes of insecticide use in agricultural landscapes, with potential implications for both pest management and conservation of non-target species.

Here, we integrate data from several national databases on the components of insecticide extent and intensity (Fig. 1) to characterize spatiotemporal patterns in bee toxic load and its components from 1997–2012 at the county scale. This time period was chosen in part for pragmatic reasons relating to data availability, but perhaps more importantly it corresponds with the window in which several significant technological and policy changes occurred in US agricultural pest management: the passage of the Food Quality Protection Act in 1996, the introduction of transgenic *Bt* crops starting in 1996, and the widespread use of neonicotinoid seed treatments starting in the mid-2000s. Our goals were to (*i*) assess trends in bee toxic load on a contact and oral basis from 1997 to 2012, nationally and for counties in the contiguous US (*ii*) identify the relative contribution of extent and intensity responsible for these changes over the same time period, and (*iii*) describe regional variation in these trends among agricultural production regions.

## Results

### Trends in bee toxic load

**Figure 2:**
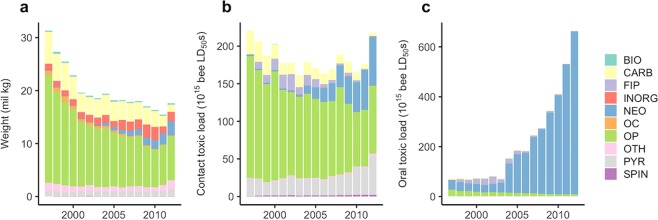
National trends in weight (**a**), and bee toxic load of all agricultural insecticides applied on a contact (**b**) and oral (**c**) toxicity basis from 1997 to 2012 by insecticide class, for the contiguous US. Mann-Kendall tests indicated a monotonic decrease in weight (tau = −0.88, *P* < 0.001), insignificant change in contact toxic load (tau = −0.27, *P* = 0.16), and a monotonic increase in oral toxic load (tau = 0.85, *P* < 0.001). Key: BIO = biological, FIP = fipronil, NEO = neonicotinoid, SPIN = spinosad, PYR = pyrethroid, CARB = carbamate, OP = organophosphate, OC = organochlorine, INORG = inorganic, and OTH = other.

**Figure 3:**
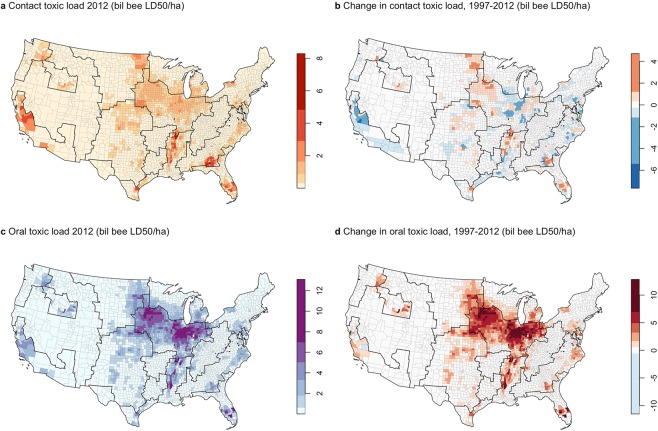
County-level patterns in contact (**a**,**b**) and oral (**c**,**d**) bee toxic load of agricultural insecticides in 2012 (**a**,**c**) and fold change from 1997–2012). Toxic load is normalized by the total area (ha) of each county, to account for variable county size. Note that California data excludes seed-applied insecticides. Color scales are based on Jenks natural breaks, which maximize similarity within classes and minimize similarity between classes, adjusted for (**b**,**d**) to center on zero. Black outlines show USDA Farm Resource Regions. Maps were created using the R statistical language version 3.6.1 (https://www.R-project.org) using the data generated in this study.

Between 1997 and 2012 bee toxic load across the contiguous US increased more than 9-fold on an oral LD_50_ basis and was roughly constant on a contact LD_50_ basis, despite an overall decline in the weight of insecticides applied (Fig. 2; note that the weight of active ingredients, not formulations, are used in our analyses). Toxic load was spatially heterogenous (Fig. 3); in 2012, the top 10% of counties accounted for 55% and 48% of contact and oral toxic load, respectively. From 1997 to 2012, oral toxic load increased in 87% of counties and decreased in 13% of counties (median change: +15-fold, IQR: +3.2-fold to +103-fold) while contact toxic load increased in 51% of counties and decreased in 49% of counties (median change: +5%, IQR: −56% to +142%).

In 2012, we estimate that agricultural insecticides were applied to ~5% of the land area of the contiguous US (26% of cropland), at an average intensity of 5 billion honey bee contact LD_50_s per treated ha and 16 billion honey bee oral LD_50_s per treated ha (Table 1).

**Table 1:** Estimates of 2012 bee toxic load and its contributors associated with insecticide use in the nine USDA Farm Resource Regions of the contiguous US.

Region	Land area (mil ha)	Extent (% ha treated)	Contact intensity (bil bee LD_50_s/treated-ha)	Oral intensity (bil bee LD_50_s/treated-ha)	Contact toxic load (bil bee LD_50_s/ha)	Oral toxic load (bil bee LD_50_s/ha)
Basin and Range*	163	0.5	5	8	0.02	0.04
Eastern Uplands	52	1.6	4	14	0.07	0.23
Fruitful Rim*	124	3.8	10	18	0.4	0.68
Heartland	73	20.2	4	21	0.79	4.22
Mississippi Portal	26	14.1	5	10	0.71	1.48
Northern Crescent	82	4.3	5	16	0.22	0.70
Northern Great Plains	75	5.6	4	12	0.23	0.68
Prairie Gateway	107	4.8	5	15	0.25	0.72
Southern Seaboard	64	4.8	7	9	0.34	0.46
Contiguous U.S.	765	5.3	5	16	0.28	0.87

*California pesticide use data excludes products applied as seed treatments so insecticide intensity and toxic load may be underestimated in these regions, which contain California counties.

### Components of bee toxic load

**Figure 4:**
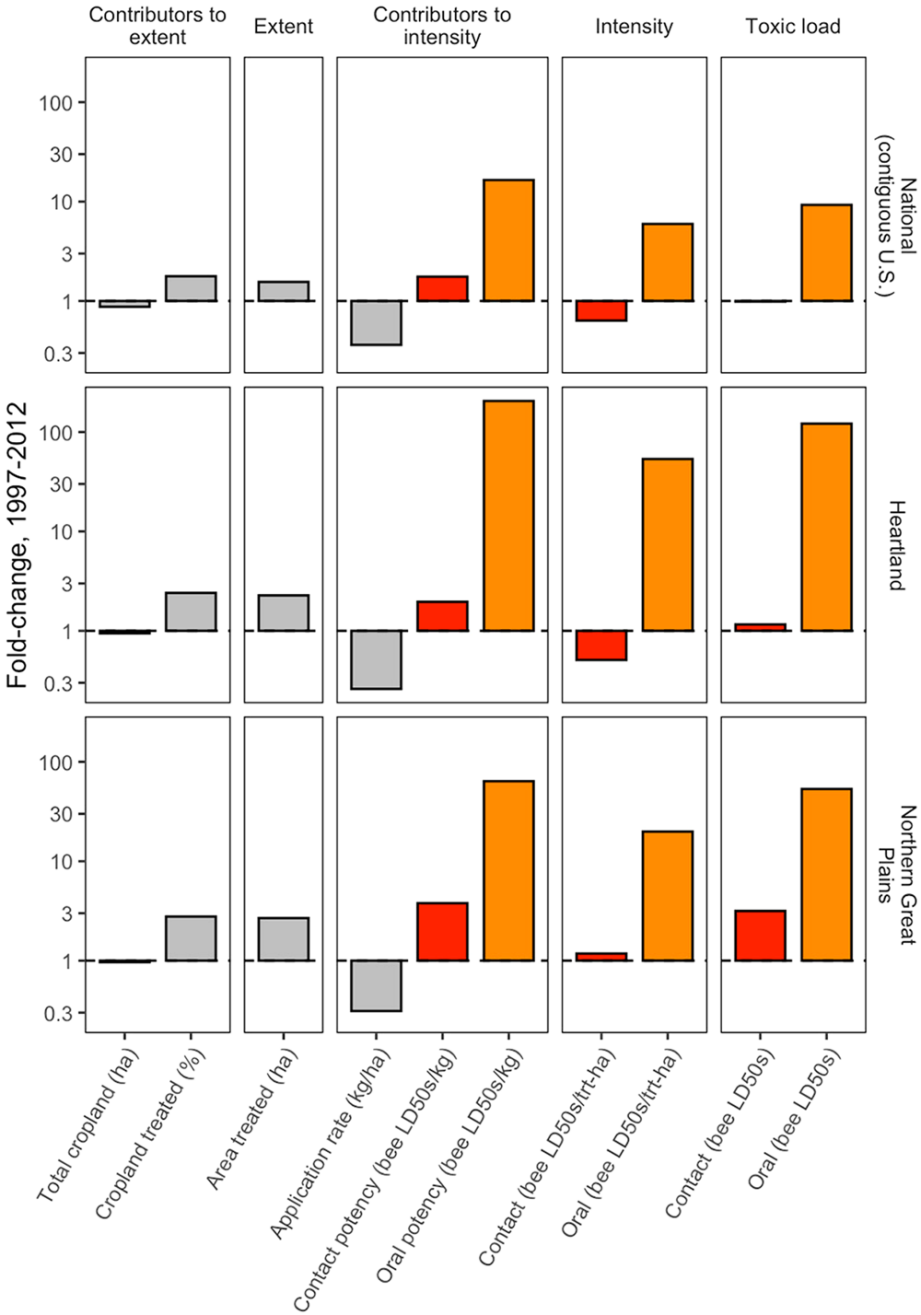
Change in the components of bee toxic load from 1997 to 2012 for the contiguous U.S. and select USDA Farm Resource Regions. Fold-change is calculated as a response ratio: Value_2012_/Value_1997_, so that a value of one represents no change, three represents a tripling, 0.3 represents a 70% decline, etc. (values are presented on a log scale). Red represents calculations from contact toxicity and orange from oral toxicity.

The main contributor to the increase in oral toxic load was insecticide intensity: a 16-fold increase in oral potency far surpassed a 64% decline in application rate (Fig. 4). The increase in insecticide potency was associated with the shifting composition of insecticide classes, away from organophosphates and toward pyrethroids and neonicotinoids (Figs. 2, S1); by 2012 neonicotinoids alone contributed 98% of oral toxic load. While contact potency also increased from 1997 to 2012 (+76%), this change was roughly counterbalanced by a corresponding decline in application rate.

Changes in extent also occurred over the study period and influenced overall toxic load; while total cropland declined slightly (−12%), the proportion of cropland treated with insecticides increased by 78%, from 15% of cropland treated in 1997 to 26% in 2012.

### Regional variation in bee toxic load and its components

**Figure 5:**
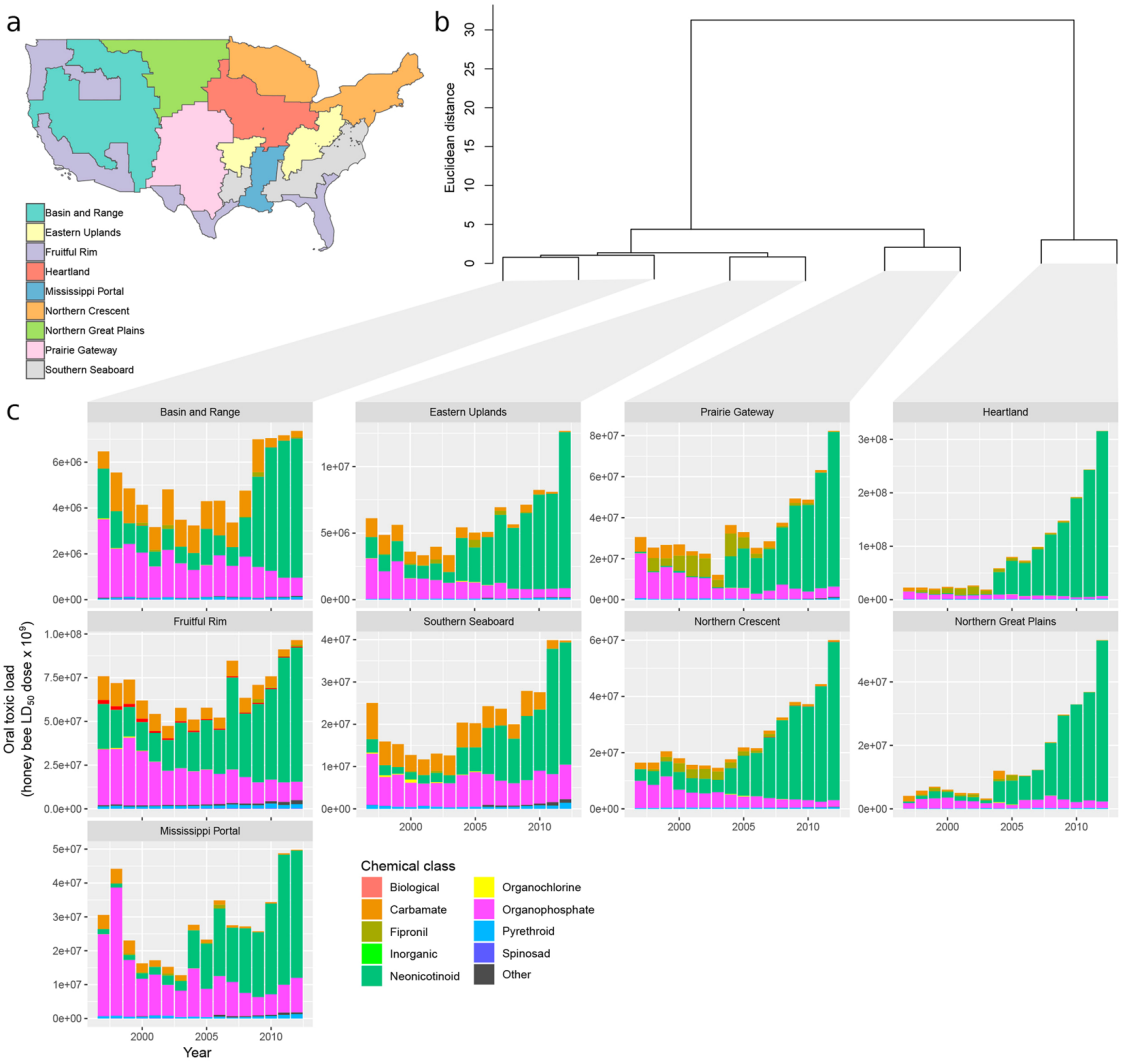
Oral toxic load by region and chemical class. Toxic load time series were constructed for each of the nine USDA Farm Resource Regions^29^ (**a**). The map was created using the R statistical language version 3.6.1 (https://www.R-project.org). Hierarchical clustering (**b**) grouped regions with similar patterns of toxic load using a Euclidean distance matrix and Ward’s linkage method. The y-axis distance between two nodes and their nearest common node is inversely proportional to similarity. Hence, the majority of variation among regions is captured in the first split that separates the Heartland and Northern Great Plains from the other seven regions. The contribution of different chemical classes to the overall toxic load pattern in each region is depicted with stacked bar plots (**c**).

The temporal dynamics of bee toxic load varied across the 9 major agricultural production regions (Figs. 3–5), which vary in crop composition (Fig. S2). Oral toxic load increased significantly in all regions except the Basin and Range (Table S1), with reductions in application rate overwhelmed by increases in potency and cropland treated (Fig. S3). The most dramatic increases occurred in the Heartland (121-fold increase) and the Northern Great Plains (53-fold increase) (Figs. 4–5). This pattern was underscored by time series cluster analysis, where the majority of total variation was captured in the node dividing the Heartland + Northern Great Plains cluster from the other seven regions (Fig. 5b). The cause of this pattern, evident when oral toxic load is parsed by chemical class, was the substantial application of neonicotinoid insecticides in the Heartland and Northern Great Plains, beginning in 2006 and increasing exponentially through 2012 (Fig. 5c). This likely reflects the dominance of corn and soybean in the Heartland and the shift to greater area under these crops in the Northern Great Plains (Fig. S2).

Conversely, changes in contact toxic load were more modest and variable, with most regions experiencing no significant trends (Table S1). The exceptions included the Eastern Uplands and Northern Crescent, where significant declines were observed (−38% and −10%, respectively), driven by application rate, and to a lesser extent, declines in total cropland (Table S1, Fig. S3). However, contact toxic load increased more than 3-fold in the Northern Great Plains (Table S1, Fig. S3), a pattern associated with a 2.7-fold increase in cropland treated and a 3.8-fold increase in contact potency (Fig. 4). In the cluster analysis, the majority of variation was captured in the node dividing the Northern Great Plains region from all other regions (Fig. S4). Notably, organophosphate toxic load increased in the Northern Great Plains from 2006 to 2012, in contrast to all other regions where the contribution of this chemical class was stable or declining (Fig. S4).

### Sensitivity analysis

We performed several analyses to characterize the robustness of our results to uncertainty in the underlying data sources and interpolation of missing values (Supplemental Material). We found that interpolation of insecticide use, crop area, and treated area in counties with missing values in our source data had only a minor effect on the overall results (Figures S5–S7). Furthermore, while the LD_50_ values for some of the insecticides used in our analysis were derived from sources with low and medium certainty, the vast majority of bee toxic load was derived from insecticides with LD_50_ values reported in high quality sources (Fig. S8). Finally, while we used the more conservative USGS ‘low’ pesticide estimate in our analyses, repeating the analyses with the ‘high’ estimate did not change the direction or significance of national or regional trends. That said, numerical estimates differed (Fig. S9, Table S2). For example, at the national scale in 2012 the high estimate was 39% and 7% greater than the low estimate for contact and oral toxic load, respectively.

## Discussion

By creating a novel dataset synthesizing land use, insecticide use, and honey bee toxicity, we characterized spatiotemporal patterns in the total inputs of insecticides to cropland in the contiguous US at the county scale. We found that bee toxic load from 1997 to 2012 was relatively constant when calculated using contact LD_50_ values, but increased nine-fold when calculated using oral LD_50_ values, with considerable variation among regions. Bee toxic load increased despite a decrease in average application rate, which emphasizes the importance of accounting for potency when assessing trends in insecticide use, as recently argued for herbicide use^25,31^. The pattern of divergence between decreasing application rate and increasing extent and potency, combined with significant variation across agricultural regions, helps to explain and reconcile the conflicting narratives of insecticide dependency that characterize recent research on US insecticide use trends^17,20–23,31^. While this analysis does not allow us to directly evaluate exposure of pollinators to insecticides, the county-level dataset provided here can be used to test relationships between insecticide use and pollinator populations and to prioritize areas for in-depth risk assessments. Furthermore, these results can inform the collection of data on insecticide use and recommendations to reduce exposure of pollinators to insecticides.

DiBartolomeis *et al*.^19^ found a greater increase in overall oral toxic load, of 48-fold compared to our calculation of 9-fold. This difference is likely due to the use of a slightly different time period, and the inclusion of an environmental half-life in the toxic load calculations, which increases the relative contribution of long-lasting insecticides. While we appreciate the value of incorporating degradation, we chose not to do so because pesticide half-lives depend on the matrix considered (e.g. soil vs. plant surface vs. water), and the pesticide source data do not include information on application method that could inform the selection of a value for half-life. For example, the Pesticide Properties Database reports a soil half-life under field conditions of 174 days for the neonicotinoid imidacloprid, while the residual half-life of the same active ingredient in the plant matrix is 4.9 days^32^. Nonetheless, it is interesting to note that the inclusion of degradation by DiBartolomeis *et al*. only magnified the trend toward increasing toxic load, suggesting that our analysis may be conservative.

We found that the increase in oral toxic load was particularly acute in the Heartland and Northern Great Plains regions, which showed a 121-fold and 53-fold increase, respectively. We attribute this pattern to the increasing use of neonicotinoid seed treatments in corn and soybean (see Figs. 2c, 5c, S9). In the Heartland, >90% of cropland (excluding pasture) in 1997 and 2012 was devoted to these two crops (Fig. S2), while the Northern Great Plains experienced significant land use change over the study period in which corn and soybean displaced other crops and conservation land^33^. Neonicotinoids accounted for the overwhelming majority of oral toxic load by 2012, and previous research^24^ showed that virtually all neonicotinoid use in corn and soybean is via seed application. These results are consistent with DiBartolomeis et al., which also found that neonicotinoid use in corn and soy was the primary driver of the observed increase in oral toxic load at the national scale.

The important role of neonicotinoid seed treatments in this analysis underscores another source of conflicting interpretations of US insecticide use trends – inconsistent or incomplete data collection. USDA started collecting data on seed treatments only in 2015^34^, and the data are not yet publicly available. The USGS Pesticide National Synthesis Project included seed-applied pesticides through 2014 (except in California), then stopped when the data provider excluded them^35^. Analyses derived from the Census of Agriculture suggest that seed treatments are at least partly captured by that dataset^22,23^, although the survey language is ambiguous^36^. Consequently, our estimate of insecticide extent is likely an underestimate. Our results show that analyses of insecticide use that do not account for seed treatments are likely missing a major component of bee toxic load, highlighting a need for more consistent and comprehensive data collection. For similar reasons, our findings on insecticide use trends for counties in California should be interpreted cautiously given the lack of seed treatment data in that state. Similarly, our results point to a need for more detailed investigation of the combined agronomic and socioeconomic drivers of neonicotinoid seed treatments, which do not seem to be cleanly related to pest pressure or field-level economics^24,30,37,38^.

Recommendations to farmers for reducing exposure of pollinators and natural enemies to insecticides have historically emphasized avoiding contact exposure, for example by spraying at night or not spraying during bloom^6^. Our finding that oral toxic load - driven by the use of systemic neonicotinoids - has surpassed contact toxic load suggests that this guidance may need to be amended. Systemic insecticides are taken up by plant tissues where they can remain active, sometimes for long periods. Indeed, a major route of exposure for foraging bees to neonicotinoids and other pesticides is through nectar and pollen obtained from flowering weeds in agricultural areas, where the weeds have taken up residues from the soil^39–41^. Seed treatments likely exacerbate this route of exposure, since neonicotinoids can remain present in the soil for years^42^; indeed, recent studies identified imidacloprid residues in plants and bee colonies, though imidacloprid use had been discontinued in these study regions in previous years^39,43^. It is sometimes possible to adjust management practices to avoid non-target exposure to systemic pesticides, but this requires longer-term planning than mitigation at the time of spraying. In apples, for example, spraying neonicotinoids 5–10 days prior to bloom ensures levels are sufficiently low in nectar and pollen to protect pollinators and natural enemies^44^. Furthermore, in a recent study of authorized uses of neonicotinoids in agricultural systems in France, in 96% of cases, an effective alternative was available, and in 78% of cases, an effective non-chemical alternative was available (such as physical or biocontrol methods), suggesting it is possible to alter practices to continue managing pests but reduce off-target effects^45^.

Importantly, our approach is not a formal risk assessment, nor does it fit neatly into existing risk assessment frameworks like that of the US-EPA^46^. The formal concept of risk–the likelihood of adverse outcome–is defined as the product of hazard and exposure. Bee toxic load corresponds to the hazard component of this definition, but we do not attempt to estimate or model pesticide fate in the environment or exposure. Formal risk assessments are also, by necessity, narrow in scope, typically evaluating single compounds with respect to specific receptor organisms over a narrow range of use scenarios. Our analysis incorporated >100 active ingredients applied in varied ways to dozens of crops, and the source data does not include information on application timing and method that would be necessary to derive a mechanistic understanding of fate and exposure. Rather, our approach integrates across compounds and use scenarios to estimate cumulative, landscape-scale insecticide hazard to bees. In this sense, we present an bee “hazard cup”, analogous to the “risk cup” paradigm used in human risk assessment^47^ and echoing Berenbaum’s^48^ extension of this paradigm to honey bee risk assessment. While there is clearly variation across insect species in toxicity, honey bees do not appear to be consistently more or less sensitive to insecticides than other insect species^49^, and so “bee toxic load” may serve as a starting point for a general assessment of the toxicity of insecticides to insect populations in a given landscape. Future extensions of this framework could seek to integrate additional layers of complexity, for example synergy between jointly applied insecticide active ingredients or between insecticides and fungicides.

The development of a county-level measure of bee toxic load, aggregated across insecticides and national in scope, advances our ability to evaluate the impacts of insecticide use on target and non-target insect populations. Previous landscape-scale studies seeking to relate insecticide use patterns to invertebrate outcomes have relied on the percentage of land in agriculture^50^, the percentage of land used to produce particular crops^51^, the percentage of land treated^22,52^, the weight of insecticides applied^53^, or weight of specific compounds^14,54^. By integrating information from national databases to summarize insecticide intensity, extent, and bee hazard (toxic load), our study provides a novel dataset that can be used in the US to investigate the potential impacts of insecticide use on diverse insect populations and dependent wildlife. Indeed, a recent study in the Netherlands created a “potential pesticide risk map” based on pesticides allowed to be applied on crops, and found a negative association between increasing pesticide toxicity in the landscape and both honey bee colony survival and wild bumble bee distributions^55^; the approach we demonstrate allows for a more empirical method for generating such maps, accounting for actual insecticide use practices, which vary regionally. While our estimates use data from 1997–2012, it is expected that the overall use patterns have remained consistent in recent years (if anything, use of neonicotinoid seed treatments in field crops has increased^56^,) and many datasets documenting insect declines encompass the time period in our study^12,13,15^. Furthermore, these national maps of bee toxic load, in combination with maps examining insect abundance and ecosystem services^57^, can be used to identify regions that should be prioritized for conservation (areas where insect abundance, diversity, or ecosystem services are high and insecticide hazard is low) or mitigation (areas where abundance of imperiled species, such as monarch butterflies, is high^58^ and insecticide hazard is also high). While there are clearly limitations to this analysis, it complements more traditional ‘bottom-up’ approaches to ecotoxicology by enabling ‘top-down’ research at the landscape scales at which insect populations and ecosystem services are structured^59^.

## Materials and Methods

This study required synthesizing data on insecticide use, land use, and toxicity from multiple national databases to generate a novel dataset documenting county-level spatiotemporal patterns in bee toxic load and its components. All data processing and analysis were performed in the R statistical language, version 3.6.1^60^. The code used to generate and analyze the dataset is documented on the following website: https://land-4-bees.github.io/bee_tox_county.

### Insecticide use data

Data on the weight of insecticides applied by county and year were downloaded from the US Geological Survey (USGS) Pesticide National Synthesis Project^61^. This dataset reports pesticide use (kg active ingredient applied in each county) for >400 of the most commonly applied herbicides, insecticides, and fungicides on agricultural crops. The estimates are derived from proprietary farmer surveys, except for California, where estimates are drawn from the state’s Department of Pesticide Regulation^61^. The dataset includes seed-applied pesticides through 2014, except in California, where they have never been included. USGS provides two pesticide estimates; we used the more conservative ‘low’ estimate (data presented in the text and figures) but repeated our analyses with the ‘high’ estimate (data presented in the Supplementary Materials) to ensure that changes over time and comparisons among regions were robust to differences in pesticide estimation. We focused on the 138 insecticides in the dataset, classified as such based on the Pesticide Properties Database (PPDB)^32^ and the Insecticide Resistance Action Committee (IRAC)^62^. Following the custom of the US Environmental Protection Agency (US-EPA) we excluded petroleum oils and distillates^4^.

### Land use data

The US Census of Agriculture is conducted every five years. We used this dataset as a source of county-level data for the years 1997, 2002, 2007, and 2012 on (*i*) cropland area treated with at least one insecticide, (*ii*) total cropland area, and (*iii*) total land area in each county. Data were downloaded from the developer page of the Quick Stats database maintained by the National Agricultural Statistics Service^63^. We focused on area in cropland rather than pastureland because USGS data indicates that only ~1% of insecticides are applied to pastureland. Missing values occurred in the dataset when data were withheld to avoid disclosing information about individual farm operations; this situation occurs mostly in cases where agricultural acreage in a county is very low. In those cases, we imputed missing values with the average of values in other years for that county if possible, or with zeros if values for the county were missing in all years^23^. Finally, counties were grouped into regions using a system developed by US Department of Agriculture (‘Farm Resource Regions’) to capture geographic variation in farming systems related to cropping patterns, similarities in soil and climate, and economic characteristics^29^.

### Toxicity data

We considered toxicity on the basis of contact and oral exposure. Contact exposure occurs when an insect encounters the insecticide on the outside of its body, for example if it is directly sprayed. Oral exposure occurs when an insect ingests the insecticide, for example when feeding on a recently treated plant. We conducted our analyses separately with each toxicity measure because of the difficulty of predicting the dominant mode of exposure from insecticide use data alone (particularly given that the data do not include method of application).

Honey bee acute toxicity values were compiled mainly from US-EPA’s ECOTOX database^64^ and the PPDB. The ECOTOX database was queried in July 2017 and values were recorded from the PPDB in June 2018. Both sources include toxicity data generated during pesticide regulatory procedures as well as from other sources (e.g. scientific journal articles). We gave preference to values generated in the regulatory processes of the US-EPA and the European Food Safety Authority (EFSA) because they tend to be generated using standardized testing protocols.

To compile honey bee LD_50_ values for the 138 insecticides present in the USGS dataset, we first searched ECOTOX for all lab-generated LD_50_ estimates for *Apis mellifera*. We then processed the data by recoding exposure types into ‘contact’, ‘oral’, or ‘other’ and standardizing units where possible into µg/bee, consulting original sources as needed to verify extreme values and to fill in missing information. The following criteria were used to select records: (*i*) exposure time 4 days or less^65^, (*ii*) contact or oral exposure, (*iii*) tests on adult bees, and (*iv*) non-zero LD_50_ estimate in units of μg/bee. We related this cleaned dataset to USGS pesticide names on the basis of CAS numbers. Similarly, we generated a dataset comprising honey bee LD_50_ values (acute contact and oral) from the PPDB, along with the source code identifying high-quality values coming from the EU regulatory process (code ‘A5’).

To generate a consensus list of contact and oral LD_50_ values for all insecticides reported in the USGS dataset (Supplemental Data 1) we used the following procedure, adapted from previous research^66^:

1. If point estimate(s) were available from US or EU regulatory bodies, we used those values, taking a geometric mean when estimates were available from both sources; otherwise,
2. If point estimate(s) were available from other sources in ECOTOX or PPDB, we used those values, taking a geometric mean when estimates were available from multiple sources; otherwise,
3. If unbounded estimate(s) were available from US or EU regulatory bodies (i.e. “greater than” or “less than” some value), we used the minimum (for “less than”) or the maximum (for “greater than”); otherwise,
4. If unbounded estimate(s) were available from other sources in ECOTOX or PPDB, we used the minimum (for “less than”) or the maximum (for “greater than”); otherwise,
5. We used the median toxicity value for the insecticide mode-of-action group, with group defined by the Insecticide Resistance Action Committee.
6. In rare cases (n = 1/138 compounds for contact toxicity and 8/138 compounds for oral toxicity) after this procedure we were still left without a toxicity estimate for a particular compound. In those cases, we used the median for all insecticides.

### Data synthesis and analysis

We synthesized the data sources described above to generate a novel dataset describing bee toxic load and its contributors (Supplemental Data 2). First, we calculated the total contact and oral bee toxic load for each county-year combination from 1997 to 2012, using the following equation adapted from previous research^25^:

1 $$Insect\,toxic\,load={\sum }_{ai=1}^{n}\frac{Weigh{t}_{ai}}{Toxicit{y}_{ai}},$$

where weight is the total weight (kg) of insecticide active ingredient (*ai*) applied in a county in a particular year, and toxicity is the contact or oral acute toxicity to honey bees (LD_50_ in μg/bee) for each *ai*. There were a minority of counties for which insecticide use data were missing in particular years. We used linear interpolation to fill in missing values for kg applied, contact toxic load, and oral toxic load. If agricultural insecticide use was never reported in a particular county (this occurred in a few, highly urban counties like New York City), we assumed it was zero.

Bee toxic load in a county is a function of the area of land treated with insecticides (*extent*) and the aggregate toxicity of the insecticides applied per unit area (*intensity*) (Fig. 1). To better elucidate bee toxic load over the study period, we calculated elements of insecticide extent and intensity for those years in which land use data were available from the agricultural census (1997, 2002, 2007, 2012). Data on county-level land use were joined with data on insecticide use and toxic load on the basis of county FIPS codes (Supplemental Data 3). Once joined, we identified and calculated the contributors to bee toxic load as defined in Fig. 1.

To test for monotonic trends in bee toxic load at the national and regional scales we used the non-parametric Mann-Kendall test. To further analyze change over time, and to compare the relative magnitude of change across indicators with disparate units, for each of our insecticide indicators we calculated fold-change from 1997 to 2012 as the response ratio: Value_2012_/Value_1997_, so that a value of one represents no change, two represents a doubling, and one half represents a decline of 50%.

To analyze regional differences in the temporal dynamics of toxic load, we performed hierarchical time series clustering using the R package ‘dtwclust’^67^. Contact and oral toxic load were calculated for years 1997–2012 each USDA Farm Resource Region. Because regions differed by as much as two orders of magnitude in absolute toxic load, we converted the absolute toxic load within each region to fold-change relative to 1997, as described above, enabling more informative clustering driven by patterns of relative toxic load. We then performed hierarchical time series clustering using a Euclidean distance matrix and Ward’s linkage method, with oral and contact toxic load analyzed separately.

## Supplementary information

Supplemental Data 1

Supplemental Data 2

Supplemental Data 3

Supplemental Data 4

Supplemental Data 5

## Data Availability

The R code and workflow associated with this paper is documented on the following website: https://land-4-bees.github.io/bee_tox_county. The datasets generated through our analyses and their associated metadata are included in Supplemental Information.

## References

[1] Scudder, G. G. In Insect Biodiversity: Science and Society (eds Foottit, R. & Adler, P.) 7-32 (Blackwell Publishing, 2009).

[2] Stork NE (2018). How many species of insects and other terrestrial arthropods are there on Earth?. Annual Review of Entomology.

[3] Pimentel D (1991). Environmental and economic effects of reducing pesticide use. BioScience.

[4] Atwood, D. & Paisley-Jones, C. Pesticide Industry Sales and Usage: 2008–2012 Market Estimates. (US Environmental Protection Agency, Washington DC, 2017).

[5] Stern VM, Smith RF, van den Bosch R, Hagen KS (1959). The Integrated Control concept. Hilgardia.

[6] Johansen CA (1977). Pesticides and pollinators. Annual Review of Entomology.

[7] Sponsler DB (2019). Pesticides and pollinators: A socioecological synthesis. Science of the Total Environment.

[8] Potts SG (2010). Global pollinator declines: trends, impacts and drivers. Trends in Ecology & Evolution.

[9] Dirzo R (2014). Defaunation in the Anthropocene. Science.

[10] Hallmann CA (2017). More than 75 percent decline over 27 years in total flying insect biomass in protected areas. PLOS ONE.

[11] Sanchez-Bayo F, Wyckhuys K (2019). Worldwide decline of the entomofauna: A review of its drivers. Biological Conservation.

[12] Cameron SA (2011). Patterns of widespread decline in North American bumble bees. Proceedings of the National Academy of Sciences.

[13] Stenoien C (2018). Monarchs in decline: a collateral landscape-level effect of modern agriculture. Insect Science.

[14] Forister ML (2016). Increasing neonicotinoid use and the declining butterfly fauna of lowland California. Biology Letters.

[15] Wepprich T, Adrion J, Ries L, Wiedmann J, Haddad N (2019). Butterfly abundance declines over 20 years of systematic monitoring in Ohio, USA. PLoS ONE.

[16] Kulhanek K (2017). A national survey of managed honey bee 2015–2016 annual colony losses in the USA. Journal of Apicultural Research.

[17] Fernandez-Cornejo, J. *et al*. Pesticide use in US agriculture: 21 selected crops, 1960-2008 (ed Economic Research Service US Department of Agriculture) (2014).

[18] Goulson D, Thompson J, Croombs A (2018). Rapid rise in toxic load for bees revealed by analysis of pesticide use in Great Britain. PeerJ.

[19] DiBartolomeis M, Kegley S, Mineau P, Radford R, Klein K (2019). An assessment of acute insecticide toxicity loading (AITL) of chemical pesticides used on agricultural land in the United States. PloS one.

[20] Benbrook CM (2012). Impacts of genetically engineered crops on pesticide use in the US - the first sixteen years. Environmental Sciences. Europe.

[21] Fausti SW (2012). Insecticide use and crop selection in regions with high GM adoption rates. Renewable Agriculture and Food Systems.

[22] Meehan, T. D. & Gratton, C. A consistent positive association between landscape simplification and insecticide use across the Midwestern US from 1997 to 2012. *Environmental Research Letters* **10**, 114001 (2015).

[23] Meehan TD, Gratton C (2016). A landscape view of agricultural insecticide use across the conterminous US from 1997 through 2012. PLOS ONE.

[24] Douglas MR, Tooker JF (2015). Large-scale deployment of seed treatments has driven rapid increase in use of neonicotinoid insecticides and preemptive pest management in US field crops. Environmental Science & Technology.

[25] Kniss AR (2017). Long-term trends in the intensity and relative toxicity of herbicide use. Nature Communications.

[26] US Environmental Protection Agency. Reregistration and other review programs predating pesticide registration review, https://www.epa.gov/pesticide-reevaluation/reregistration-and-other-review-programs-predating-pesticide-registration (2017).

[27] Sanchez-Bayo, F. Insecticides mode of action in relation to their toxicity to non-target organisms. *Journal of Environmental & Analytical Toxicology* **S4**, 002 (2012).

[28] Sparks TC (2013). Insecticide discovery: an evaluation and analysis. Pesticide Biochemistry and Physiology.

[29] Heimlich, R. Farm Resource Regions. (U.S. Department of Agriculture, Economic Research Service, 2010).

[30] Papiernik SK, Sappington TW, Luttrell RG, Hesler LS, Allen KC (2018). Overview: Risk factors and historic levels of pressure from insect pests of seedling corn, cotton, soybean, and wheat in the United States. Journal of Integrated Pest Management.

[31] National Academies of Sciences, E. & Medicine. Genetically engineered crops: experiences and prospects. (National Academies Press, 2016). 28230933

[32] University of Hertfordshire. Pesticide Properties Database, http://sitem.herts.ac.uk/aeru/ppdb/en/ (2017).

[33] Otto CR, Roth CL, Carlson BL, Smart MD (2016). Land-use change reduces habitat suitability for supporting managed honey bee colonies in the Northern Great Plains. Proceedings of the National Academy of Sciences.

[34] US Department of Agriculture, E. R. S. ARMS Farm Financial and Crop Production Practices, https://www.ers.usda.gov/data-products/arms-farm-financial-and-crop-production-practices/ (2019).

[35] US Geological Survey. Pesticide national synthesis project, annual pesticide use maps: 1992-2012, https://water.usgs.gov/nawqa/pnsp/usage/maps/index.php (2019).

[36] US Department of Agriculture, N. A. S. S. Census of Agriculture Report Form Guide, https://www.nass.usda.gov/AgCensus/Report_Form_and_Instructions/2012_Report_Form/2012_RFG_Final.pdf (2012).

[37] Mourtzinis S (2019). Neonicotinoid seed treatments of soybean provide negligible benefits to US farmers. Scientific reports.

[38] Bredeson MM, Lundgren JG (2015). Thiamethoxam seed treatments have no impact on pest numbers or yield in cultivated sunflowers. J. Econ. Entomol..

[39] Botías C, David A, Hill EM, Goulson D (2016). Contamination of wild plants near neonicotinoid seed-treated crops, and implications for non-target insects. Science of the Total Environment.

[40] Long EY, Krupke CH (2016). Non-cultivated plants present a season-long route of pesticide exposure for honey bees. Nature Communications.

[41] McArt SH, Fersch AA, Milano NJ, Truitt LL, Böröczky K (2017). High pesticide risk to honey bees despite low focal crop pollen collection during pollination of a mass blooming crop. Scientific Reports.

[42] Jones A, Harrington P, Turnbull G (2014). Neonicotinoid concentrations in arable soils after seed treatment applications in preceding years. Pest Manag. Sci..

[43] Woodcock BA (2017). Country-specific effects of neonicotinoid pesticides on honey bees and wild bees. Science.

[44] Biddinger DJ, Rajotte EG (2015). Integrated pest and pollinator management—adding a new dimension to an accepted paradigm. Current Opinion in. Insect Science.

[45] Jactel H (2019). Alternatives to neonicotinoids. Environment International.

[46] US EPA. Guidance for Assessing Pesticide Risks to Bees. (ed Office of Pesticide Programs) (Washington, DC, 2014).

[47] US Congress. In *Public Law* 104–170 (ed. 104th Congress) (1996).

[48] Berenbaum MR (2015). Does the honey bee “risk cup” runneth over? Estimating aggregate exposures for assessing pesticide risks to honey bees in agroecosystems. Journal of Agricultural and Food Chemistry.

[49] Hardstone MC, Scott JG (2010). Is *Apis mellifera* more sensitive to insecticides than other insects?. Pest Manag. Sci..

[50] Smart, M. D., Pettis, J. S., Euliss, N. & Spivak, M. S. Land use in the Northern Great Plains region of the US influences the survival and productivity of honey bee colonies. *Agriculture, Ecosystems & Environment* **230**, 139–149 (2016).

[51] Huseth AS, Chappell TM, Chitturi A, Jacobson AL, Kennedy GG (2018). Insecticide resistance signals negative consequences of widespread neonicotinoid use on multiple field crops in the US cotton belt. Environmental Science & Technology.

[52] Gibbs KE, Mackey RL, Currie DJ (2009). Human land use, agriculture, pesticides and losses of imperiled species. Diversity and Distributions.

[53] Benton TG, Bryant DM, Cole L, Crick HQ (2002). Linking agricultural practice to insect and bird populations: a historical study over three decades. Journal of Appled Ecology.

[54] McArt SH, Urbanowicz C, McCoshum S, Irwin RE, Adler LS (2017). Landscape predictors of pathogen prevalence and range contractions in US bumblebees. Proceedings of the Royal Society B: Biological Sciences.

[55] Yasrebi-de Kom, I. A., Biesmeijer, J. C. & Aguirre-Gutiérrez, J. Risk of potential pesticide use to honeybee and bumblebee survival and distribution: A country-wide analysis for The Netherlands. *Diversity and Distributions* (2019).

[56] Tooker, J. F., Douglas, M. R. & Krupke, C. H. Neonicotinoid seed treatments: limitations and compatibility with integrated pest management. *Agricultural & Environmental Letters* **2** (2017).

[57] Koh I (2016). Modeling the status, trends, and impacts of wild bee abundance in the United States. Proceedings of the National Academy of Sciences of the United States of America.

[58] Tracy JL, Kantola T, Baum KA, Coulson RN (2019). Modeling fall migration pathways and spatially identifying potential migratory hazards for the eastern monarch butterfly. Landscape Ecology.

[59] Beketov MA, Liess M (2012). Ecotoxicology and macroecology–time for integration. Environmental Pollution.

[60] R: A Language Environment for Statistical Computing, version 3.6.1, http://www.R-project.org (R Foundation for Statistical Computing, Vienna, 2019).

[61] Baker, N. T. & Stone, W. W. Estimated annual agricultural pesticide use for counties of the conterminous United States, 2008-12. Report No. 907, (US Geological Survey, Reston, VA, 2015).

[62] IRAC. The IRAC Mode of Action Classification, https://www.irac-online.org/modes-of-action/ (2019).

[63] USDA NASS. Quick Stats 2.0, http://quickstats.nass.usda.gov (2018).

[64] US EPA. ECOTOX Knowledgebase, https://cfpub.epa.gov/ecotox/ (2017).

[65] Sanchez-Bayo F, Goka K (2014). Pesticide Residues and Bees – A Risk Assessment. PLoS ONE.

[66] Nowell LH, Norman JE, Moran PW, Martin JD, Stone WW (2014). Pesticide toxicity index—a tool for assessing potential toxicity of pesticide mixtures to freshwater aquatic organisms. Science of the Total Environment.

[67] dtwclust: Time Series Clustering Along with Optimizations for the Dynamic Time Warping Distance, https://CRAN.R-project.org/package=dtwclust (2019).

