# Supplementary material for: County-level analysis reveals a rapidly shifting landscape of insecticide hazard to honey bees (Apis mellifera) on US farmland
Source: Sci Rep. 2020 Jan 21;10:797. doi: 10.1038/s41598-019-57225-w (PMC6972851; doi:10.1038/s41598-019-57225-w)
Supplement: Supplementary file 5 — Supplemental Data 5 [file 41598_2019_57225_MOESM5_ESM.pdf]

SUPPLEMENTAL MATERIAL

Douglas, M.R, Sponsler, D.B., Lonsdorf, E.V. and C.M. Grozinger. County-level analysis reveals a rapidly shifting landscape of insecticide hazard to honey bees (*Apis mellifera*) on US farmland.

Sensitivity analysis

We performed several analyses to characterize the robustness of our results to uncertainty in the underlying data sources. One source of uncertainty in our estimates stemmed from interpolating missing values for insecticide use, crop area, and treated area in counties with missing values in our source data. However, the sensitivity analysis revealed that interpolation had a minor effect on estimates of bee toxic load (Figures S5-S7). At the national scale, pesticide use was interpolated for fewer than 2% of counties in each year, and these counties contributed less than 0.05% to weight applied, contact toxic load, and oral toxic load. Interpolation was more frequent for treated cropland; nonetheless cropland treated was interpolated for < 6% of county-year combinations and these interpolated values contributed < 1% to total cropland treated. Across data items, interpolation tended to be rare in the regions driving our results (Heartland, Northern Great Plains), and more common in regions with generally little cropland (e.g. Basin and Range; Figures S5-S7).

Another source of uncertainty in our analyses concerned the honey bee LD_{50} values underlying the translation of insecticide use into bee toxic load. For each US county, we calculated the percentage of bee toxic load contributed by compounds with ‘high’, ‘medium’, and ‘low’ uncertainty in their LD_{50} values. Uncertainty was considered low if LD_{50} values were derived from US or EU regulatory procedures, medium if LD_{50} values were compound-specific but derived from other scientific sources, and high if LD_{50} values were estimated by a median value for the class or insecticides as a whole. In each year of the dataset active ingredients with high-quality values (those generated as part of the US or EU regulatory processes) accounted for the vast majority of insecticide weight (79-86%), contact toxic load (91-98%), and oral toxic load (98-100%; Figure S7). Low-quality values (those missing and so estimated from class medians) contributed modestly to insecticide weight (1.5-15%) but very little to contact toxic load (< 1%) and oral toxic load (< 3%), likely reflecting that active ingredients without honey bee toxicity data are more likely to be found in classes with generally low toxicity.

To test the sensitivity of our findings to different methods of insecticide estimation, we repeated our main analyses using the less-conservative USGS ‘high’ estimates of insecticide use (Figures S3, S8, S9; Table S2). Qualitative patterns in bee toxic load and its components were extremely similar for the two estimates, supporting our conclusions regarding overall trends and regional variability in insecticide indicators. However, there were quantitative differences. For example, the 2012 estimate of bee toxic load at the national scale based on the ‘high’ USGS estimate was 39% higher on a contact-toxicity basis and 7% higher on an oral-toxicity basis than the equivalent value using the ‘low’ estimate (Table S2).

SUPPLEMENTAL FIGURES

Figure S1. Honey bee LD_{50} values by insecticide class. Key: FIP = fipronil, NEO = neonicotinoid, SPIN = spinosad, PYR = pyrethroid, CARB = carbamate, OP = organophosphate, OC = organochlorine, INORG = inorganic, BIO = biological, and OTH = other.

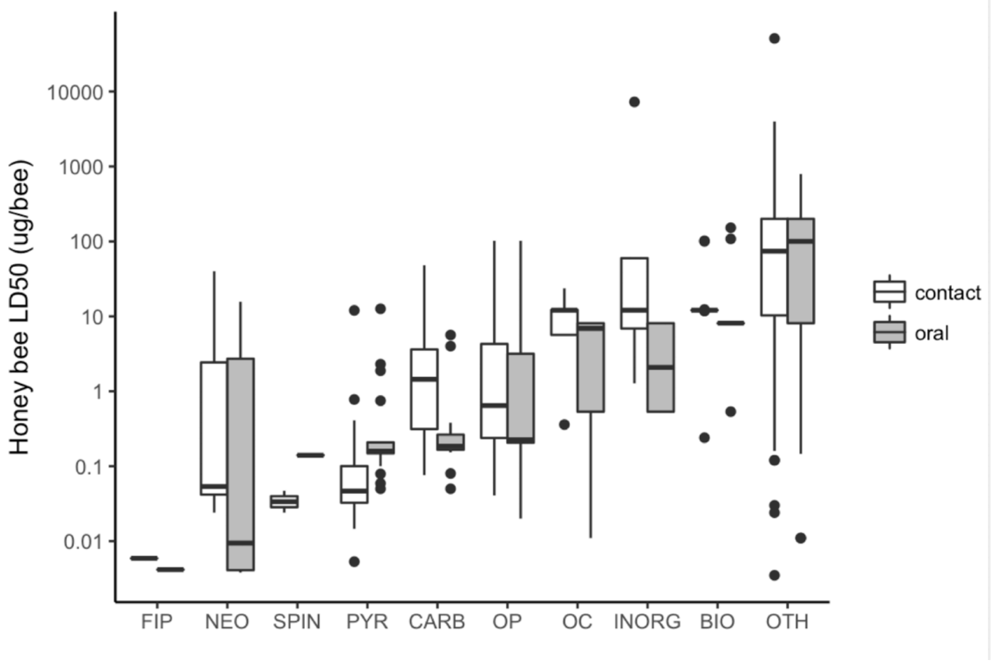

Figure S2. Composition of cropland by region in 1997 and 2012, based on crop area data from the U.S. Census of Agriculture.

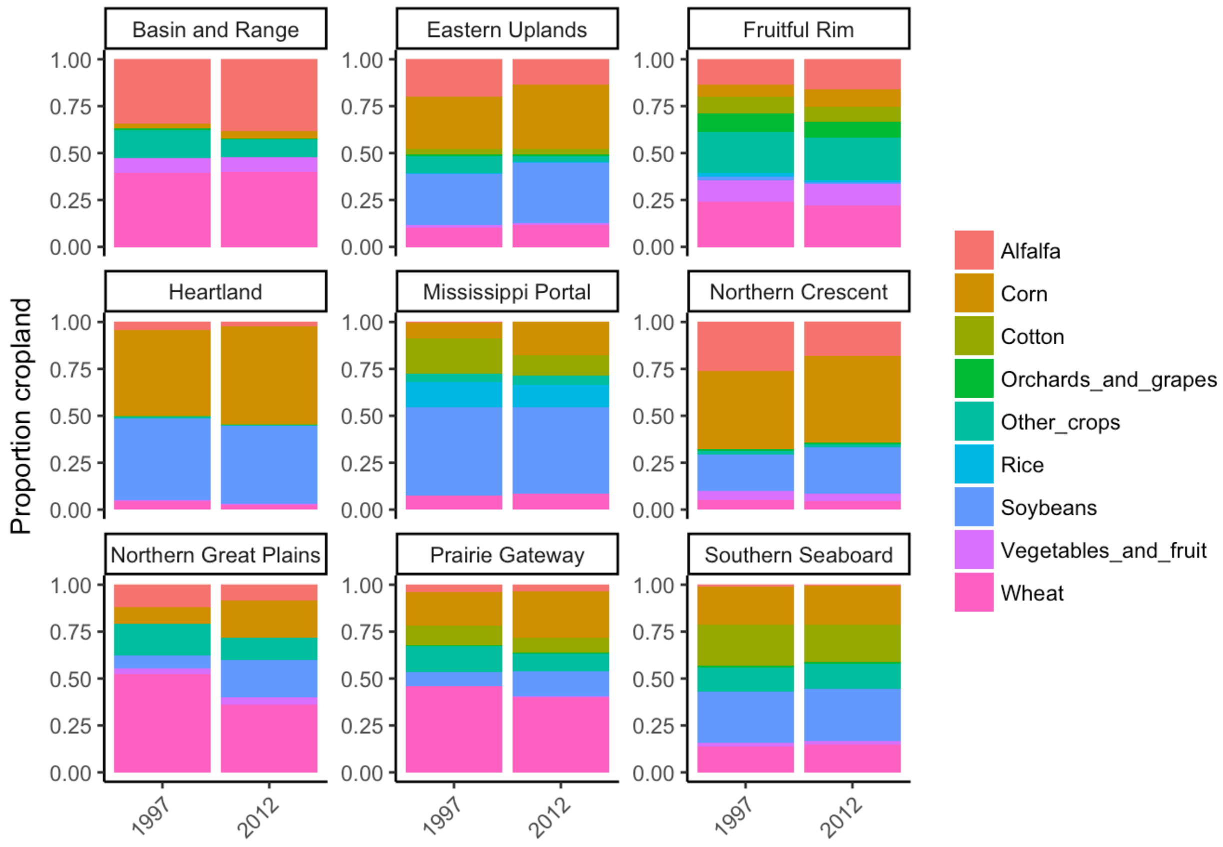

Figure S3. Change in the components of bee toxic load from 1997 to 2012 for all agricultural production regions, based on the USGS low (left) and high (right) pesticide use estimates. Fold-change is calculated as a response ratio: $Value_{2012}/Value_{1997}$, so that a value of one represents no change, two represents a doubling, one half represents a decline of 50% (values are presented on a log scale).

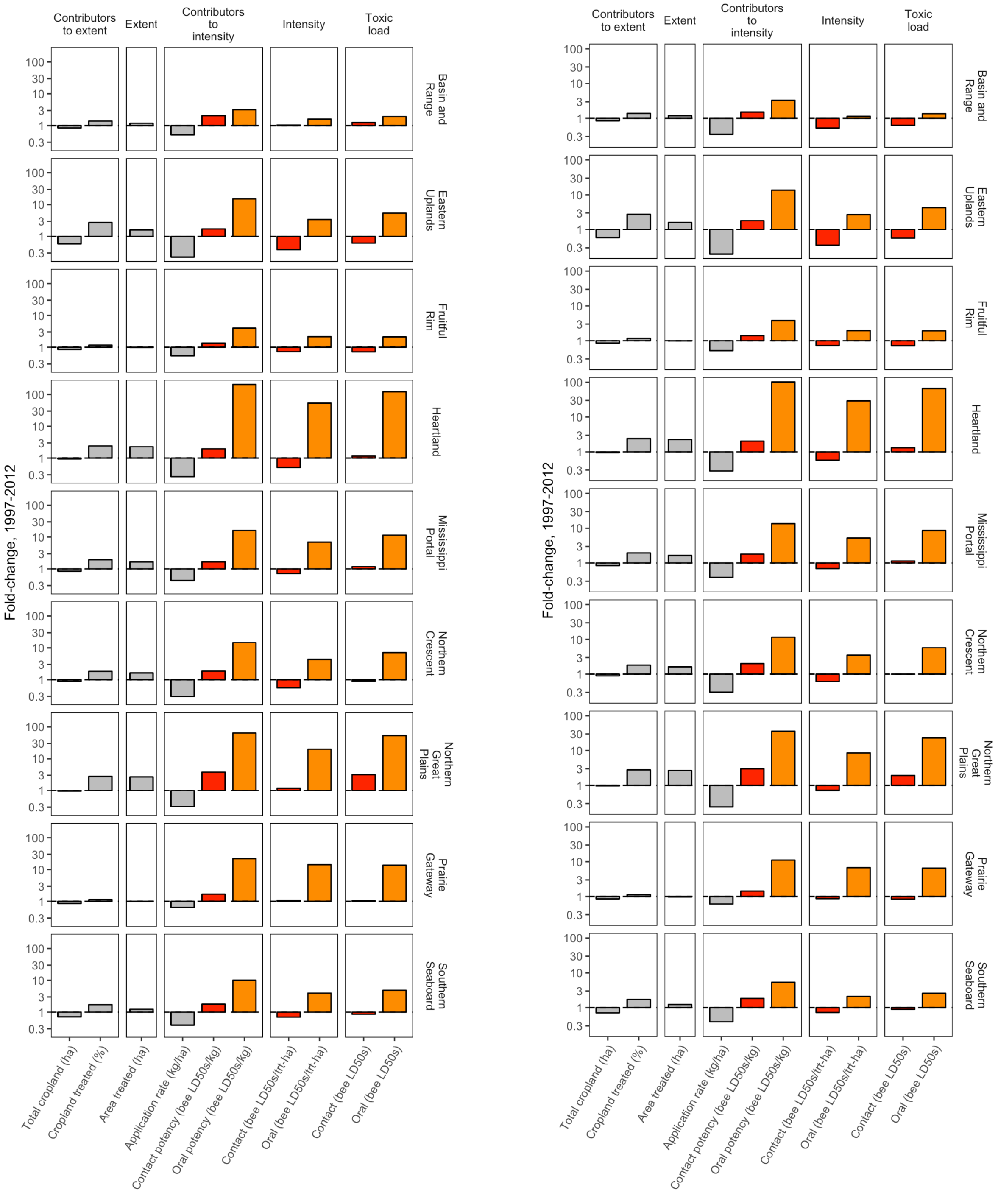

Figure S4. Contact toxic load by region and chemical class. Toxic load time series were constructed for each of the nine USDA Farm Resource Regions. Hierarchical clustering (left) grouped regions with similar patterns of toxic load using a Euclidean distance matrix and Ward's linkage method. The y-axis distance between two nodes and their nearest common node is inversely proportional to similarity. Hence, the majority of variation among regions is captured in the first split that separates the Northern Great Plains from the other eight regions. The contribution of different chemical classes to the overall toxic load pattern in each region is depicted with stacked bar plots (right).

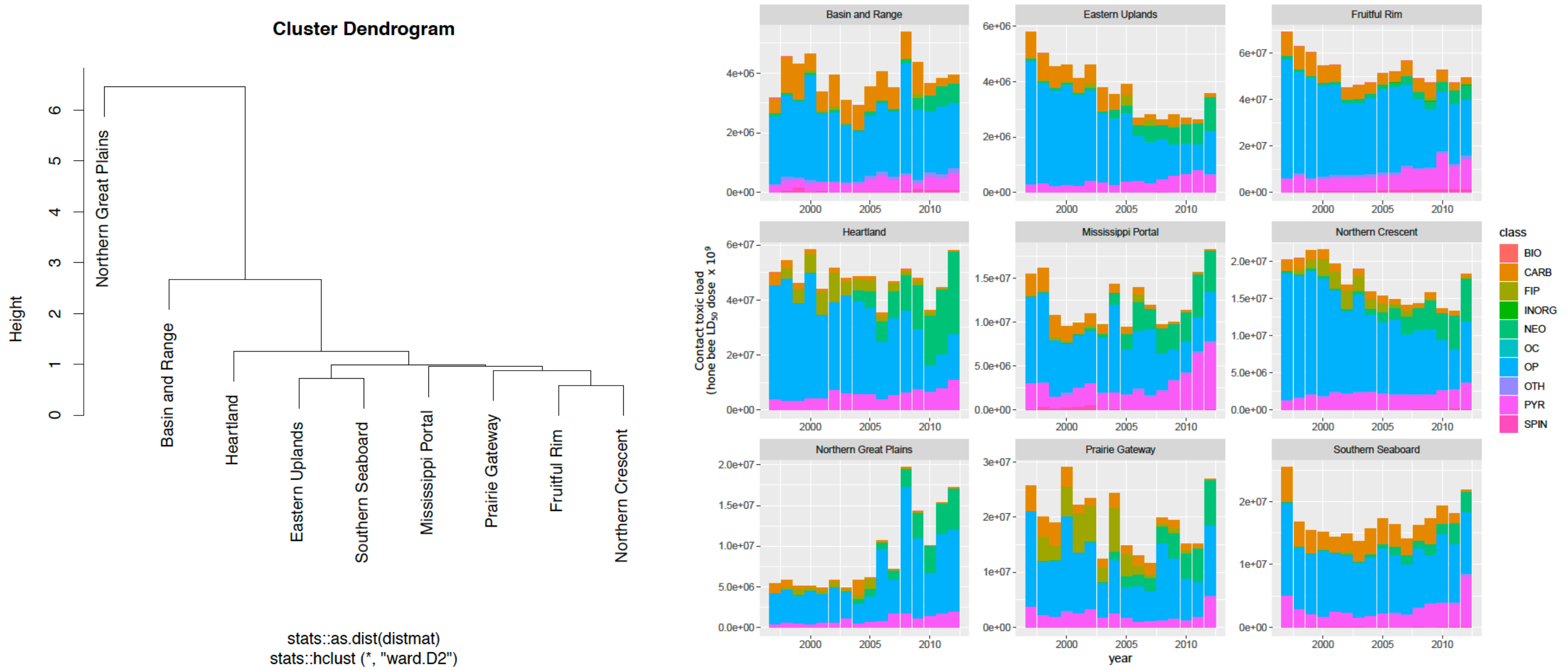

Figure S5. Contribution of counties with interpolated insecticide values to total counties (n_interp), mass applied (kg_low), contact toxic load (ct_tox), and oral toxic load (or_tox) for each of nine agricultural regions and all regions combined.

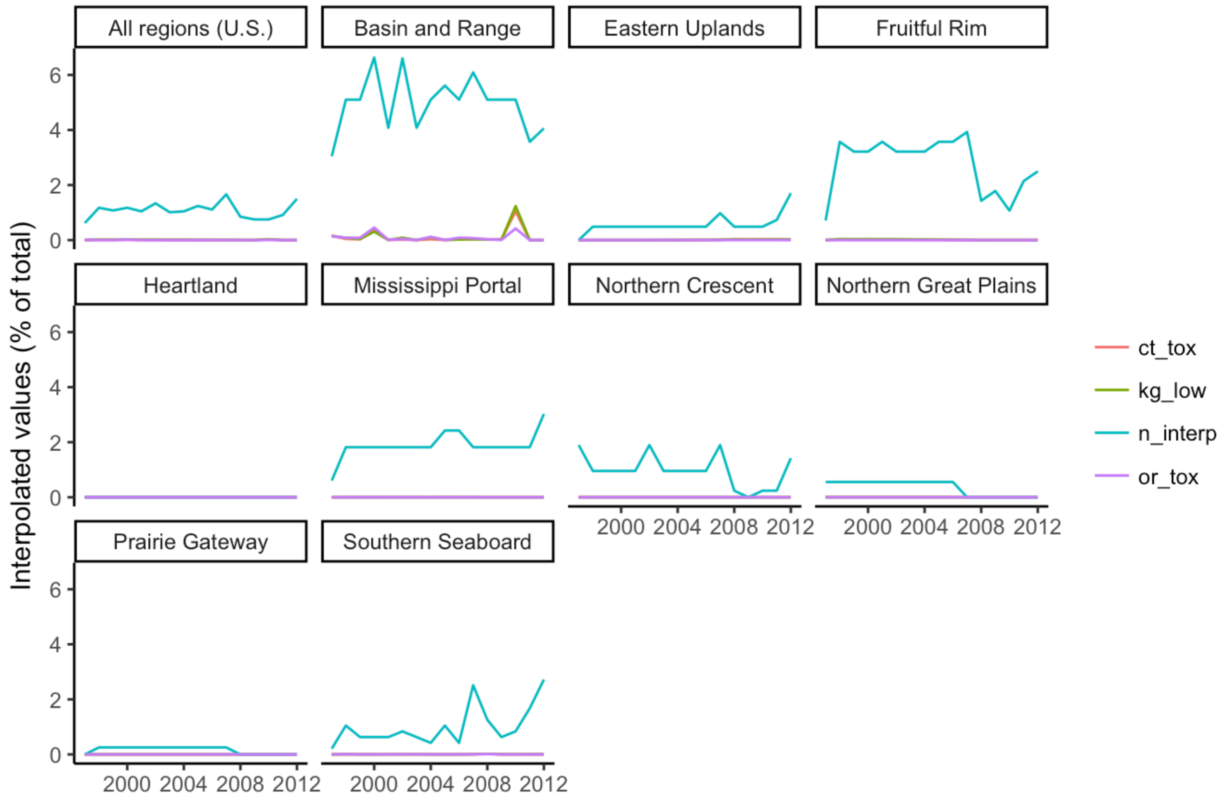

Figure S6. Contribution of counties with interpolated cropland area values to total counties (n_interp) and cropland (crop_ac) for each of nine agricultural regions and all regions combined.

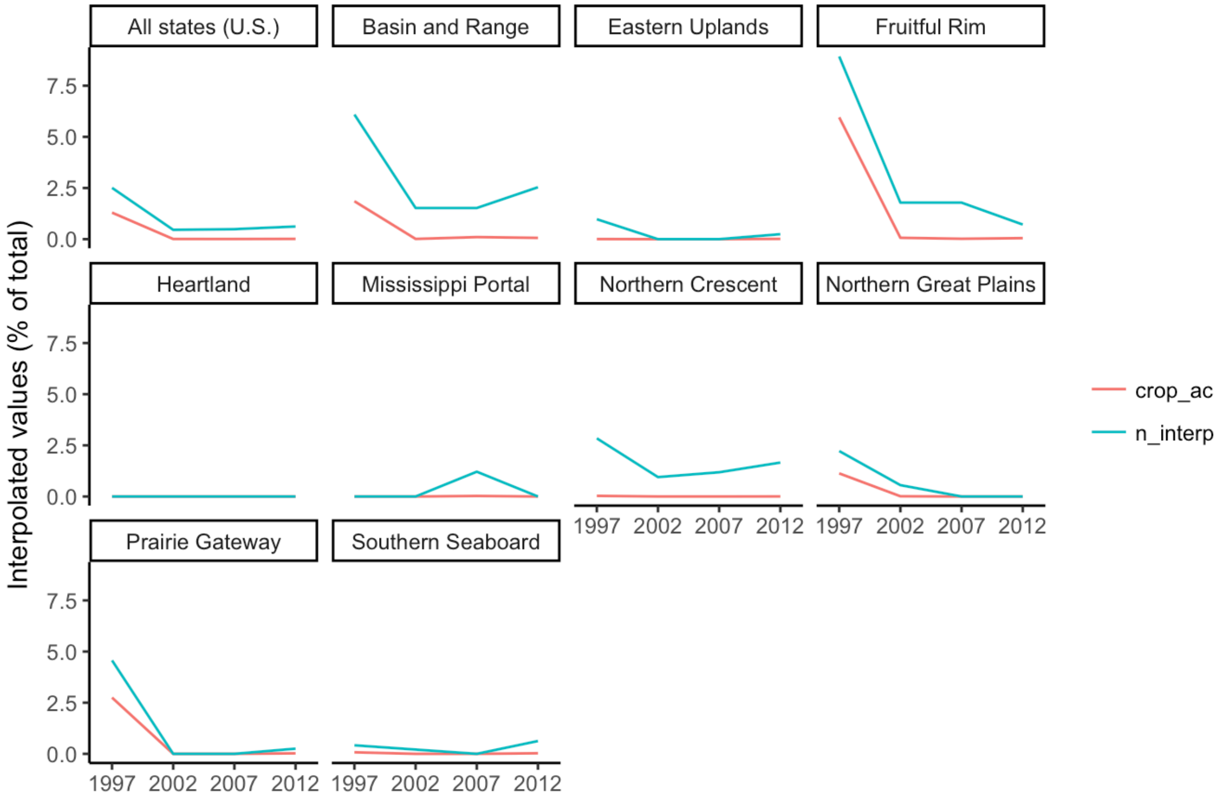

Figure S7. Contribution of counties with interpolated treated area values to total counties (n_interp) and treated cropland (trt_ins_ac) for each of nine agricultural regions and all regions combined.

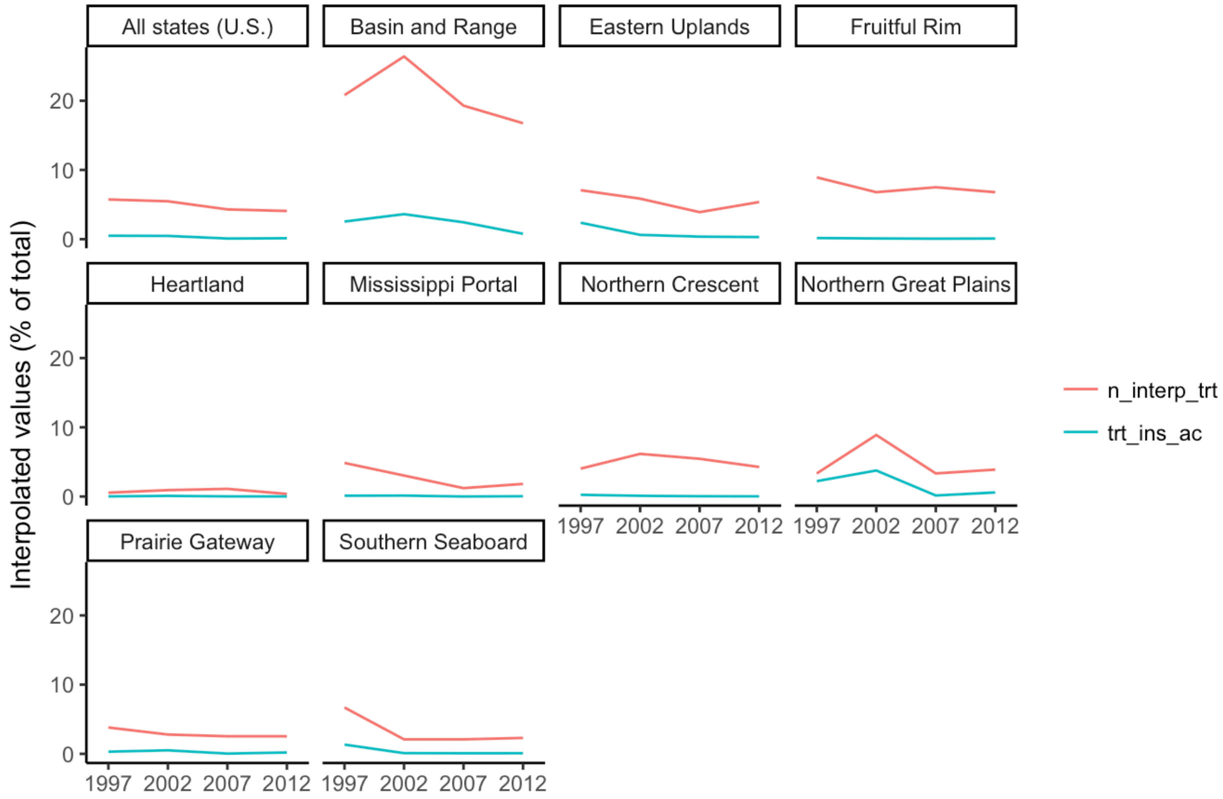

Figure S8. Contribution of LD_{50} values of low, medium, and high uncertainty to national estimates of bee toxic load. LD_{50} values are considered low-uncertainty if they are derived from US or EU regulatory procedures, medium-uncertainty if they are compound-specific but derived from the general scientific literature, and high-uncertainty if they are based on class or insecticide median values.

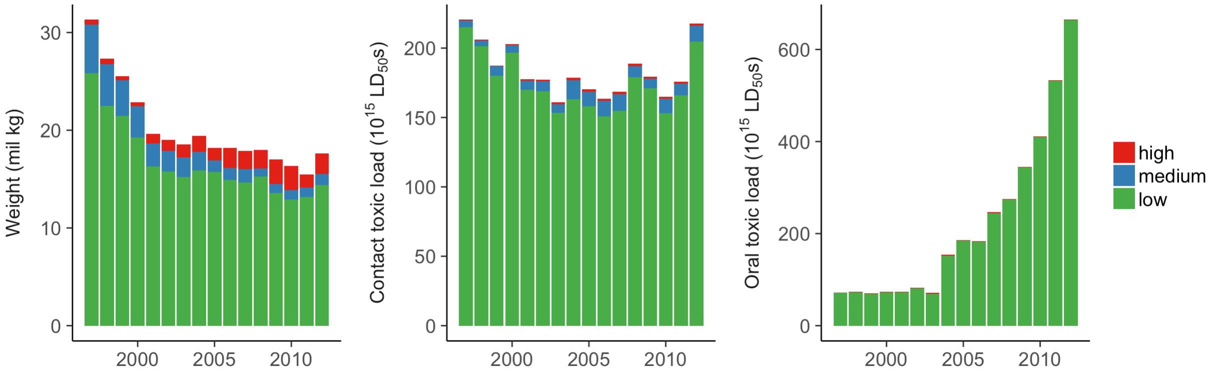

Figure S9. National analysis repeated with the USGS 'high' insecticide use estimate.

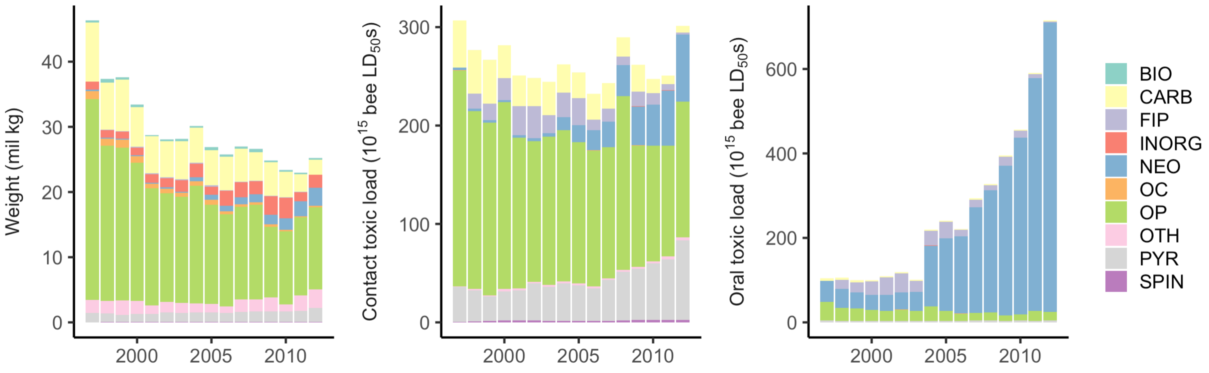

Table S1. Results from Mann-Kendall trend tests for contact-based and oral-based bee toxic load, from 1997-2012 for nine US agricultural production regions. Tests were considered significant at $P < 0.05$.

	Contact toxic load		Oral toxic load	
Region	tau	*P*	tau	*P*
Basin and Range*	0.07	0.75	0.30	0.12
Eastern Uplands	**-0.72**	**< 0.001**	**0.67**	**< 0.001**
Fruitful Rim*	-0.37	0.053	**0.58**	**0.002**
Heartland	-0.20	0.30	**0.92**	**< 0.001**
Mississippi Portal	0.10	0.62	**0.78**	**< 0.001**
Northern Crescent	**-0.65**	**< 0.001**	**0.88**	**< 0.001**
Northern Great Plains	**0.62**	**0.001**	**0.83**	**< 0.001**
Prairie Gateway	-0.22	0.26	**0.78**	**< 0.001**
Southern Seaboard	0.27	0.16	**0.68**	**< 0.001**
Contiguous U.S.	-0.27	0.16	**0.85**	**< 0.001**

* California pesticide use data excludes products applied as seed treatments so insecticide toxic load may be underestimated in these regions, which contain California counties.

Table S2. Estimates of 2012 bee toxic load and its contributors for agricultural production regions of the U.S. associated with insecticide use, based on the less-conservative 'high' estimate from the USGS National Pesticide Synthesis Project.

Region	Land area (mil ha)	Extent (% ha treated)	Contact intensity (bil bee LD_{50}s/ treated-ha)	Oral intensity (bil bee LD_{50}s/ treated-ha)	Contact toxic load (bil bee LD_{50}s/ ha)	Oral toxic load (bil bee LD_{50}s/ ha)
Basin and Range*	163	0.5	8	10	0.04	0.05
Eastern Uplands	52	1.6	8	17	0.13	0.27
Fruitful Rim*	124	3.8	11	19	0.43	0.71
Heartland	73	20.2	5	22	1.10	4.39
Mississippi Portal	26	14.1	7	11	0.94	1.58
Northern Crescent	82	4.3	8	18	0.35	0.78
Northern Great Plains	75	5.6	7	13	0.39	0.75
Prairie Gateway	107	4.8	8	17	0.37	0.84
Southern Seaboard	64	4.8	11	11	0.52	0.52
**Contiguous U.S.**	765	5.3	7	18	0.39	0.93

* California pesticide use data excludes products applied as seed treatments so insecticide intensity and toxic load may be underestimated in these regions, which contain California counties
